# Early Initiation of Ceftaroline-Based Combination Therapy for Methicillin-resistant Staphylococcus aureus Bacteremia

**DOI:** 10.21203/rs.3.rs-4095478/v1

**Published:** 2024-03-15

**Authors:** Addison S. Hicks, Mackenzie A. Dolan, Megan D. Shah, Sarah E. Elwood, James A. Platts-Mills, Gregory R. Madden, Zachary S. Elliott, Joshua C. Eby

**Affiliations:** University of Virginia Health System

**Keywords:** methicillin-resistant Staphylococcus aureus bacteremia, combination therapy, vancomycin, daptomycin, ceftaroline

## Abstract

**Purpose:**

Monotherapy with vancomycin or daptomycin remains guideline-based care for methicillin-resistant *Staphylococcus aureus* bacteremia (MRSA-B) despite concerns regarding efficacy. Limited data support potential benefit of combination therapy with ceftaroline as initial therapy. We present an assessment of outcomes of patients initiated on early combination therapy for MRSA-B.

**Methods:**

This was a single-center, retrospective study of adult patients admitted with MRSA-B between July 1, 2017 and April 31, 2023. During this period, there was a change in institutional practice from routine administration of monotherapy to initial combination therapy for most patients with MRSA-B. Combination therapy included vancomycin or daptomycin plus ceftaroline within 72 hours of index blood culture and monotherapy was vancomycin or daptomycin alone. The primary outcome was a composite of persistent bacteremia, 30-day all-cause mortality, and 30-day bacteremia recurrence. Time to microbiological cure and safety outcomes were assessed. All outcomes were assessed using propensity score-weighted logistic regression.

**Results:**

Of 213 patients included, 118 received monotherapy (115 vancomycin, 3 daptomycin) and 95 received combination therapy with ceftaroline (76 vancomycin, 19 daptomycin). The mean time from MRSA-positive molecular diagnostic blood culture result to combination therapy was 12.1 hours. There was no difference between groups for the primary composite outcome (OR 1.58, 95% CI 0.60, 4.18). Time to microbiological cure was longer with combination therapy (mean difference 1.50 days, 95% CI 0.60, 2.41). Adverse event rates were similar in both groups.

**Conclusions:**

Early initiation of ceftaroline-based combination therapy did not improve outcomes for patients with MRSA-B in comparison to monotherapy therapy.

## Background

*Staphylococcus aureus* bacteremia is associated with a high mortality rate with a risk for metastatic complications(1). Independent factors associated with poor outcomes include complicated infections, persistent bacteremia, treatment failure, and incomplete source control(2,3). In addition, mortality from methicillin-resistant *Staphylococcus aureus* bacteremia (MRSA-B) is higher than mortality from methicillin-susceptible *S. aureus* (MSSA) and has driven a search for alternative therapies(4,5).

The elevated mortality rate observed for MRSA-B may be attributable, in part, to the standard therapy, vancomycin or daptomycin(6). Vancomycin is not as effective as β-lactam antibiotics for treatment of MSSA(7), which could be extrapolated to the treatment of MRSA(8). A study by Turnidge et al. identified treatment of *S. aureus* bacteremia (independent of methicillin-resistance) with vancomycin (as compared to a β-lactam) as a risk factor for 30-day morality(9). There are concerns regarding slow bacterial killing by vancomycin, reduced *S. aureus* susceptibility to vancomycin, and failures with daptomycin(10–12). Multiple studies of β-lactam combination therapy for MRSA-B have been performed with some showing an overall benefit of combination therapy on mortality and bacterial clearance but also potential for higher rate of toxicity compared with vancomycin or daptomycin monotherapy(4,11,13,14).

A growing body of evidence supports the use of ceftaroline in combination with vancomycin or daptomycin for MRSA-B(12,15,16). Unlike some β-lactam antibiotics assessed for synergy with vancomycin and daptomycin, ceftaroline has direct anti-MRSA activity. In addition, combination vancomycin plus ceftaroline allows for enhanced binding and cell wall thinning with improved drug penetration, and limits vancomycin sequestration within the cell wall(17). A potential mechanism for synergy between daptomycin and ceftaroline includes changes in cell membrane surface charge, where an increase in net surface negativity promotes a more favorable binding site of the positively charged daptomycin complex(17). *In vitro* data have demonstrated the synergistic activity of vancomycin or daptomycin with ceftaroline by showing an inverse correlation between the minimum inhibitory concentration (MIC) of vancomycin or daptomycin and ceftaroline, supporting the ‘seesaw-effect’ hypothesis(18–20). Furthermore, time-kill studies have demonstrated improved bacterial eradication with combination therapy(18–20).

Combination therapy with vancomycin or daptomycin plus ceftaroline has been used as salvage therapy for MRSA-B in patients with persistent bacteremia and monotherapy failure. These studies have demonstrated rapid clearance of bacteremia after switching to combination therapy(2,21–23). As combination therapy may clear bacteremia rapidly, and persistent bacteremia is associated with increased mortality(24,25), analyzing the effect of early initiation of combination therapy rather than salvage therapy for MRSA-B is warranted.

Two studies evaluating early initiation of combination therapy for MRSA-B have been performed. McCreary et al. retrospectively evaluated patients receiving combination daptomycin-ceftaroline (DAP-CPT) at any time during treatment for at least 72 hours to standard of care (SOC) monotherapy. While they did not observe a statistically significant difference in 30-day mortality overall or in the subgroup of DAP-CPT started early (within 72 hours of index culture) compared to SOC, they did observe a non-significant reduction in 30-day mortality by 80% in an additional subgroup of patients with a primary endovascular source of infection who had been started on DAP-CPT early within 72 hours of index culture versus SOC(15). Early combination therapy was not the target of the primary analysis, making up less than 50% of the cohort. Geriak et al. randomized patients to receive early DAP-CPT treatment versus SOC monotherapy with vancomycin, and stopped the study early after noticing a significant difference in in-hospital mortality of 0% (0/17 patients) in the DAP-CPT group and 26% (6/23) in the SOC group(16). While the findings were promising, the study was initially designed to assess duration of bacteremia rather than mortality, and was at risk for bias due to unbalanced randomization and early termination(26).

Preclinical and limited clinical data suggest that there may be a role for ceftaroline-based combination therapy for MRSA-B. There remain limited data for assessing the effect of early combination therapy for MRSA-B with subsequent de-escalation to monotherapy which holds promise to maximize efficacy while minimizing toxicity. The primary objective of this study was to evaluate clinical outcomes for patients with MRSA-B initiated on early combination therapy with vancomycin or daptomycin plus ceftaroline in comparison to vancomycin or daptomycin monotherapy.

## Methods

### Study Design and Population

This was a retrospective cohort study conducted at University of Virginia (UVA) Health evaluating hospitalized adult patients with MRSA-B who received combination therapy with vancomycin or daptomycin plus ceftaroline or monotherapy with vancomycin or daptomycin within 72 hours of index blood culture collection between July 1, 2017 and April 31, 2023 for their first episode of MRSA-B during the study period. Patients escalated from monotherapy to combination therapy beyond 72 hours of index blood culture were analyzed as part of the monotherapy group. Patients were excluded if they had polymicrobial blood cultures, had a prior episode of MRSA-B within the preceding 90 days, were cultured and/or treated for MRSA-B at an outside facility prior to transfer (due to lack of access to these records), had initial therapy started at least 5 days after index blood culture, died or were transitioned to hospice care before speciation of index blood cultures, lacked one or more repeat blood cultures within 96 hours of the index culture, or were discharged against medical advice. The research protocol was approved by the Institutional Review Board for Health Sciences Research at UVA.

Prior to this study, Infectious Diseases (ID) consultation was made mandatory for patients with MRSA-B, with ID fellow physicians being paged directly by the microbiology laboratory with positive MRSA results from a rapid molecular diagnostic assay performed on blood cultures(27). This system has resulted in rapid initiation of MRSA-directed therapy.

In March 2020, there was a general change in practice accompanied by an internal consensus statement among providers in UVA’s ID Division favoring early initiation of ceftaroline-containing combination therapy for MRSA-B. The statement does allow for initial monotherapy at the discretion of the treating ID physician in those with presumed primary line source of infection, prompt line removal, and judgement that bacteremia is likely uncomplicated. The statement also leaves the agent used in combination with ceftaroline up to this physician. The internal consensus document also recommended de-escalation of combination therapy to monotherapy after clinical improvement and negative follow-up blood cultures at 96 hours. While monotherapy was permitted by this statement, combination therapy was the default initial treatment recommendation made by fellows prior to completing the mandatory consultation when automatically paged by the laboratory about a positive MRSA rapid molecular diagnostic result in the blood.

### Data Collection

We identified all hospitalized adult patients with at least one positive blood culture for MRSA during the study period, followed by manual chart review within the electronic medical record to collect additional data. Data collected included patient demographics, severity of illness, source and foci of infection, pursuit of source control, COVID status, comorbidities (including diabetes mellitus, CKD/ESRD, cirrhosis, and immunocompromised status) and Charlson comorbidity index(CCI)(28), and treatment characteristics. Severity of illness was assessed using the quick Pitt bacteremia score (qPitt)(29), with the highest values for each component part occurring 24 hours before and after index blood culture being recorded. Based on the literature, source of infection was categorized as primary, endovascular; secondary, non-endovascular; and catheter-related, with patients who had bacteremia of unclear source categorized as primary, endovascular(15,16,30,31). Additional foci of infection including catheter, vertebral bone/joint, nonvertebral bone/joint, SSTI/surgical, endovascular, respiratory (including septic pulmonary emboli), and hardware were also collected(16). A patient was judged to have received source control for group balancing if source control was determined to be unattainable or not required, was fully obtained successfully, or was partially obtained (which would include control of some but not all foci of infection).

The target dose of daptomycin was at least 8 mg/kg every 24 hours and ceftaroline was 600 mg every 8 hours, with renal dose adjustments as indicated. Vancomycin was dosed by an ID-trained pharmacist targeting trough levels between 15–20 mcg/mL and monitored after discharge by ID-trained pharmacists as part of an outpatient parenteral antimicrobial therapy (OPAT) program. Area under the curve (AUC) dosing was not performed at our institution during this time period. Antimicrobial susceptibility testing was performed by Vitek^®^ 2, except for ceftaroline, which was performed via E-test.

### Outcomes and Definitions

The primary outcome was a composite of persistent bacteremia, 30-day all-cause mortality, and 30-day bacteremia recurrence. Persistent bacteremia was defined as a positive blood culture at least 7 days after index blood culture without microbiological cure, 30-day all-cause mortality as death within 30 days of the index positive blood culture, and 30-day bacteremia recurrence as isolation of MRSA from blood cultures within 30 days of microbiological cure.

Secondary outcomes included time to microbiological cure, hospital length of stay, 30-day readmission, and 90-day bacteremia recurrence. Time to microbiological cure was defined as the number of days between index blood culture collection and the first negative blood culture without subsequent blood culture growth within 72 hours. Thirty-day readmission included infection- or antibiotic-related readmission within 30 days of discharge from the hospital admission associated with the MRSA-B episode. Adverse drug events (ADEs) were also evaluated. Acute kidney injury (AKI) was defined as serum creatinine increase by ≥ 0.3 mg/dL within 48 hours or ≥ 1.5x baseline within the last 7 days in non-hemodialysis patients during treatment of MRSA-B(32). AKI was further staged for severity by serum creatinine criterion outlined in the KDIGO guidelines(32). Neutropenia was defined as an absolute neutrophil count < 1000 cells/mm^3^ and eosinophilia was absolute eosinophil count > 500 cells/mcL. Elevated creatine phosphokinase (CPK) was defined as > 1000 U/L or 5x upper limit normal, and myalgias and rash were recorded if noted by an ID clinician. Patients were considered immunocompromised if they had: human immunodeficiency virus, solid tumor, hematologic malignancy, solid organ transplant, hematologic transplant, or ongoing immunosuppressive therapy.

### Statistical Analysis

Analyses were performed using R, version 4.0 (R Core Team, Vienna, Austria). To estimate the association between combination therapy and all primary and secondary outcomes, both unweighted and propensity score-weighted logistic regression were performed. First, propensity scores were estimated using a logistic regression model, with combination therapy as the outcome and covariates including age, gender, weight, hospital location, immunocompromised status, diabetes, chronic or end-stage renal disease, CCI, indwelling hardware, presence of a catheter, infection site, source control status, qPitt score, altered mental status, respiratory rate, hypotension or use of vasopressors, receipt of other anti-MRSA therapy, time from collection to first antibiotic administration, and an interaction between year and COVID status. Final model selection was determined by model fit using the Akaike information criterion. Propensity scores were then calculated as $\frac{1}{\text{probability of group assignment}}$ for each group, stabilized by dividing by the mean weight, and winsorized to the 1st and 99th percentiles. The primary, secondary and adverse event outcomes were modeled using logistic regression for dichotomous outcomes and linear regression for continuous outcomes with therapy group assignment as the only predictor and weights incorporated using *svyglm* from the *survey* package in R. Using the same methodology, a post-hoc sensitivity analysis was performed using a 48-hour cutoff for inclusion in the combination therapy group. Patients previously included in the combination therapy group that were started on combination therapy between 48- and 72-hours of index blood culture collection were re-classified as monotherapy.

## Results

### Patient Characteristics

**Figure 1:**
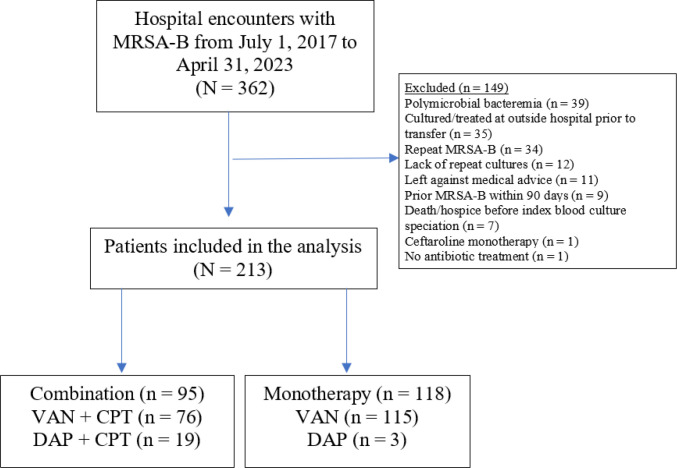
Study population and analysis cohort Abbreviations: CPT, ceftaroline; DAP, daptomycin; MRSA-B, Methicillin-resistant *Staphylococcus aureus* bacteremia; VAN, vancomycin

We identified 362 hospital encounters with MRSA-B during the study period, of which 149 encounters were excluded (Fig. 1). The most common reasons for exclusion were polymicrobial bacteremia, culture and/or treatment for MRSA-B at an outside facility prior to transfer, and prior or repeat episodes of MRSA-B within the study period. Therefore, 213 patients met inclusion criteria, of whom 118 patients received monotherapy and 95 patients received combination therapy (Fig. 1). ID consult is mandatory for MRSA-B at our institution, which in practice translated to the involvement of an ID physician in the care of 99.1% (211/213) of patients included in this study.

In the unweighted analysis, more patients in the combination therapy group had a qPitt score ≥ 2 (as well as SBP < 90 mmHg/vasopressor use and RR > 25 per min/requiring mechanical ventilation) within 24 hours of index blood culture and were more likely to have a respiratory focus of infection (Table 1). The monotherapy group contained a greater proportion of immunocompromised patients. The most common foci of infection were skin and soft tissue infection/surgical and endovascular for both groups, although there were more patients in the monotherapy group with an endovascular infection. Source control (including partial source control) was achieved in 89% of patients overall. The two treatment arms were more closely balanced after incorporation of propensity score weights.

Twelve patients in the study were found to be COVID positive during their hospitalization for MRSA-B. The overall incidence of COVID in the study was 5.6% (12/213), but was 9.9% (12/121) for patients admitted after 2019. By treatment group, 1 patient (0.85%) was COVID positive in the monotherapy group and 11 patients (11.6%) were positive in the combination therapy group.

### Time to Antibiotics and De-escalation

Of the 118 patients in the monotherapy group, 115 patients initially received vancomycin and 3 patients received daptomycin. Of these patients, 21.2% of them received monotherapy after the March 2020 consensus statement. The mean time from index blood culture collection to initial anti-MRSA antibiotic was 10.4 hours, with 10 (8.5%) patients receiving an anti-MRSA antibiotic before index culture collection (Table 1). Fifteen patients (13%) were escalated to combination therapy with ceftaroline at a mean of 5.4 days. Six of these 15 patients were subsequently de-escalated to monotherapy.

Of the 95 patients who received ceftaroline-based combination therapy, 76 patients initially received combination with vancomycin and 19 patients received combination with daptomycin. Of these patients, 97.9% of them received combination therapy after the March 2020 consensus statement. The mean time to the first anti-MRSA antibiotic was 4.8 hours with 13 (13.7%) patients receiving an anti-MRSA antibiotic before index blood culture collection (Table 1). The mean time from MRSA result identified by rapid diagnostic assay to the start of combination therapy was 12.1 hours. The mean time from index blood culture collection to the start of combination therapy was 33.2 hours. Nineteen patients (26%) who initially received vancomycin plus ceftaroline were switched to daptomycin plus ceftaroline after a mean of 5.8 days. The mean duration of combination therapy was 15.2 days, while the median duration was 8 days.

There were 110 patients who received combination therapy at any point during their treatment course and 70 (64%) were de-escalated to monotherapy (6/15 in the monotherapy group and 64/95 in the combination therapy group). The mean time to de-escalation was 10.4 days. The most common reasons for de-escalation to monotherapy included blood culture clearance with source control and clinical improvement (n = 41), consolidation of regimen at discharge (n = 15), and cytopenia (n = 2). Other reasons included cost, deep-seated infection ruled out, and switch to alternative antibiotics.

### Primary Outcomes

In the unweighted analysis, 29 (24.5%) patients in the monotherapy group and 41 (43%) in the combination group met the primary composite outcome of persistent bacteremia, 30-day all-cause mortality, and 30-day bacteremia recurrence (OR 2.23 for combination therapy, 95% CI 1.24, 4.01) (Table 2). When propensity weights were assigned, there was no difference between groups for the primary composite outcome (OR 1.58 for combination therapy, 95% CI 0.60, 4.18). Among the component parts of the propensity-weighted primary outcome, there were greater odds of persistent bacteremia for combination therapy patients (OR 4.28, 95% CI 1.64, 11.19) and no difference between groups for 30-day mortality (OR 1.00, 95% CI 0.33, 3.08) or 30-day recurrence (too few patients to calculate an OR) (Table 2).

### Secondary Outcomes

At 3 days after the start of therapy, 49 (52%) patients and 91 (77%) patients in the unweighted combination therapy and monotherapy groups, respectively, had negative blood cultures. In the propensity-weighted analysis, time to microbiological cure was longer in the combination therapy group (mean difference 1.50 days, 95% CI 0.60, 2.41) as was hospital length of stay (mean difference 13.38 days, 95% CI 2.43, 24.34) (Table 3). A post-hoc sensitivity analysis excluding 12 outlier admissions with LOS 1.5 times the inter quartile range above the 3rd quartile continued to show a statistically significant longer LOS for combination therapy (mean difference 3.49, 95% CI 0.05, 6.93). Thirty-day readmission was not different between groups in the propensity score-weighted logistic regression (OR 0.74, 95% CI 0.35, 1.54).

### Subgroup Analyses

We attempted to identify subgroups of patients for which combination therapy may improve outcomes in comparison to monotherapy, hypothesizing that those with greater risk of adverse outcome may show benefit from combination therapy. A high-risk subgroup analysis was performed for patients with a qPitt score > 2 within 24-hours of index culture draw. qPitt score was excluded from the propensity weighting model for this analysis. For the primary composite outcome, this demonstrated an unweighted OR 2.15 (95% CI 0.92, 5.04), and continued to show no difference between groups when propensity weights were assigned (OR 1.09, 95% CI 0.25, 4.74). Among the component parts of the primary outcome, there were greater odds of persistent bacteremia (OR 7.02, 95% CI 1.61, 30.61) and no difference between groups for 30-day mortality (OR 0.58, 95% CI 0.13, 2.66) or 30-day recurrence (too few patients to calculate an OR). There was a significantly longer hospital length of stay (mean difference 23.63 days, 95% CI 1.24, 46.02) for combination therapy patients in this group and no difference between time to microbiologic cure (mean difference 1.08 days, 95% CI −0.50, 2.65) or 30-day readmission (OR 0.95, 95% CI 0.27, 3.35). High-risk subgroup analyses were also performed for patients aged > 65 and patients with CCI > 4 which did not demonstrate any difference in the primary composite outcomes comparing combination therapy and monotherapy.

### Safety Outcomes

Safety outcomes were assessed for patients who had available data. Overall, there were no significant differences between the groups (Table 4). The most common ADE was AKI in both groups with 28/71 patients (39%) in the combination therapy group and 38/104 patients (37%) in the monotherapy group experiencing AKI (OR 1.49, 95% CI 0.60, 3.34). More patients demonstrated KDIGO stage 3 AKI in the combination therapy group (OR 2.69, 95% CI 0.60, 12.13). Eosinophilia also occurred slightly more frequently in the combination therapy group (OR 1.45, 95% CI 0.66, 3.19). Overall, 9 patients in each group (9% combination therapy group, 8% monotherapy group) experienced an ADE that led to an antibiotic change or discontinuation (OR 1.10, 95% CI 0.36, 3.32).

### Sensitivity Analysis

Hypothesizing that the 72-hour cutoff for inclusion in the combination therapy group could introduce bias by allowing clinical decline or repeat positive cultures to influence the decision to start combination therapy, a post hoc sensitivity analysis was performed to evaluate a 48-hour cutoff. This analysis included 132 patients in the monotherapy group and 81 in the combination therapy group. Fourteen patients were re-classified from combination therapy to monotherapy with this new cutoff. For the primary composite outcome, this change demonstrated an unweighted OR 2.19 (95% CI 1.22, 3.95) comparing combination therapy to monotherapy, and continued to show no difference between groups when propensity weights were assigned (OR 1.14, 95% CI 0.43, 3.04). The component parts of the primary outcome showed a greater odd of persistent bacteremia (OR 3.96, 95% CI 1.39, 11.22) and no difference between groups for 30-day mortality (OR 0.70, 95% CI 0.23, 2.14) or 30-day recurrence (too few patients to calculate an OR). There was no difference between any secondary outcomes in the propensity score-weighted logistic regression: time to microbiologic cure (mean difference 0.69 days, 95% CI −0.23, 1.88), hospital length of stay (mean difference 4.26 days, 95% CI −8.74, 17.27), and 30-day readmission (OR 1.03, 95% CI 0.46, 2.30).

## Discussion

MRSA-B is associated with significant morbidity and mortality and current therapies are suboptimal. Evidence suggests a benefit of using ceftaroline-based combination therapy for MRSA-B. This is the largest study to date to investigate early initiation of combination therapy with ceftaroline compared to early monotherapy with vancomycin or daptomycin. Our institution is able to make changes in initial therapy for MRSA-B since the ID fellow is notified as soon as positive blood cultures show *S. aureus* by a rapid molecular diagnostic assay(27).

We found no difference between monotherapy and early combination therapy in our primary composite outcome, which consisted of persistent bacteremia, 30-day all-cause mortality, and 30-day bacteremia recurrence. The lack of mortality benefit contrasts with findings from the small prospective study by Geriak et al.(16,26).

Our finding of no mortality difference may be due to most patients in our study receiving vancomycin plus ceftaroline, whereas other studies evaluating early combination therapy investigated daptomycin plus ceftaroline(15,16). It is likely that many of our patients received this combination regimen due to already receiving vancomycin monotherapy at time of notifying the ID fellow of MRSA-B, who then recommended the addition of ceftaroline.

The mean time to microbiological cure was 4.38 days in the weighted combination group versus 2.71 days in the weighted monotherapy group (mean difference 1.50 days, 95% CI 0.60, 2.41) based on the defined initiation of combination therapy within 72 hours of index blood culture collection. This finding contrasts with the CAMERA-1 study(33) and there are reasons to consider that unmeasured confounding may contribute to our finding. Therefore, we performed a post-hoc sensitivity analysis redefining groups by initiation of combination therapy with a 48-hour cutoff rather than a 72-hour cutoff, leaving less time for clinical decline or repeat positive cultures to influence a switch to combination therapy. With this shorter cutoff, there was no longer a significantly longer time to microbiologic cure, which is in-line with prior literature suggesting quicker clearance of bacteremia and is consistent with residual confounding in the 72-hour cutoff group(2,21–23). There was still more persistent bacteremia in the combination therapy group.

There are several potential causes of residual confounding. Use of combination therapy was ultimately up to the provider who may have been influenced by clinical severity or repeat positive blood culture within 72 hours. Longer time to microbiologic cure in the combination therapy group may also have been impacted by differences in the frequency of obtaining repeat blood cultures throughout the study timeframe, with an institutional effort during the latter half of the timeframe to obtain repeat cultures less frequently. Our institution transitioned to a new blood culture machine with presumed greater sensitivity for detecting growth resulting in measurement of additional culture-positive days in the latter half of the study when combination was more frequently chosen than monotherapy.

Hospital length of stay was longer in the combination therapy group (mean difference 13.38 days, 95% CI 2.43, 24.34). This finding persisted despite excluding outlier admissions with LOS 1.5 times the inter quartile range above the 3rd quartile range (mean difference 3.49, 95% CI 0.05, 6.93), suggesting that outliers affecting the mean was not the driving force behind longer LOS in the combination therapy group. It’s possible this is a true effect, but it may also be related to the effects of COVID on global hospital LOS in the latter half of the study period (thus disproportionately affecting the combination therapy group) or to some residual imbalance between groups.

We also performed a high-risk subgroup analysis looking specifically at patients with more severe illness at the time of their index culture draw as indicated by qPitt bacteremia score > 2, hypothesizing that those with more severe illness may benefit from early initial combination therapy. This subgroup was chosen as the qPitt score has been shown to be a good predictor of mortality in *Staphylococcus aureus* bacteremia(28), and the information contained within the score is more likely to be readily available for early treatment decision making than source of infection. Within this subgroup, there was still no difference in the primary outcome (OR 1.09, 95% CI 0.25, 4.74) to suggest a benefit for choosing combination therapy for these patients. Similarly, there was no demonstrated benefit for subgroups including patients aged > 65 years or with CCI > 4.

We did not observe any statistically significant differences in safety outcomes between combination therapy and monotherapy groups (Table 4). AKI was the most common ADE and overall rate of AKI was similar between the two groups. Although there was not a statistically significant increased odds of AKI in the combination group compared to monotherapy group, there is still a concern that combination therapy with vancomycin and possibly daptomycin with β-lactam antibiotics could be associated an increased risk of AKI in comparison to monotherapy. A large well-designed randomized trial may unmask this risk of AKI as was performed in the CAMERA-2 study(4).

Limitations of our investigation include a small sample size, though larger than other studies of early combination therapy, and retrospective design. Despite use of propensity-weighted regression, we observed some residual imbalance in the baseline characteristics, including a higher proportion of primary bacteremia and higher qPitt score (particularly in the component parts of hypotension/vasopressor use and tachypnea/mechanical ventilation) in the combination therapy group that could have masked a benefit of combination therapy. The change in practice to combination therapy occurred close in time to the onset of the COVID-19 pandemic which led to changes in many aspects of the American healthcare system, including changes in antibiotic utilization, access to care, outcomes, and chronic disease burden(34–37). Thus, there may be unmeasured changes in practice or the healthcare system that affected outcomes in this study. Of note, data on therapeutic drug monitoring was not collected, although an ID-trained pharmacist was included in managing vancomycin and daptomycin in all cases of MRSA bacteremia and did so with consistent methodology. Importantly, as with the use of any antimicrobial, while the ability to measure collateral damage is limited, the potential damage itself is presumed to be greater when more antimicrobials are utilized simultaneously. This is of particular concern in this study, as both the mean (15.2 days) and median (8 days) durations of combination therapy were greater than the 96 hours recommended for consideration of de-escalation to monotherapy in the consensus document.

In this retrospective study, there was no clear benefit of early initiation of combination therapy for MRSA-B, despite having a population with high severity of illness and burden of infection as well as the ability to start combination therapy early based on paging of molecular diagnostic results. While propensity-matched retrospective studies are subject to some confounding, any undetected effect on mortality and benefit to bacterial clearance (if present) of combination therapy is likely not clinically substantial and must be considered in the context of collateral damage associated with the use of additional antibiotics. This includes increased potential for the development of antimicrobial resistance, *C. difficile* infection, and other ADEs. With specific attention to ADEs and antimicrobial stewardship, additional prospective studies of initial combination therapy and comparison of ceftaroline-based combination regimens may be warranted.

## Figures and Tables

**Table 1:** Characteristics at time of index blood culture by treatment group, with and without propensity score weighting

Characteristic	Combination^a^ (n = 95)	Monotherapy^a^ (n = 118)	Combination^a^ (weighted)	Monotherapy^a^ (weighted)

Age, years	58.66 + 1.49	62.96 + 1.28	59.74 + 1.73	61.01 + 1.50

Female	34 (35.8)	39 (33.1)	34 (34.3)	35 (34.4)

Weight, kg	84.84 + 2.66	87.41 + 2.51	88.21 + 4.12	87.08 + 2.43

Comorbidities	45 (47.4)	48 (40.7)	46 (45.7)	46 (45.4)
Diabetes	34 (35.8)	34 (28.8)	32 (31.7)	31 (30.7)
CKD/ESRD	9 (9.5)	11 (9.3)	8 (7.5)	10 (9.7)
Cirrhosis	9 (21)	33 (28)	17 (17.4)	23 (23)
Immunocompromised				

Charlson comorbidity index^b^	3.09 + 0.20	2.87 + 0.20	2.98 + 0.23	2.93 + 0.20

Located in ICU at time of index blood culture	22 (23.2)	23 (19.5)	23 (23.3)	23 (22.8)

qPitt ≥ 2	47 (49.5)	42 (35.6)	44 (43.5)	40 (39.7)

Temperature < 36°C	10 (10.5)	9 (7.6)	11 (10.6)	12 (11.4)

SBP < 90 mmHg or vasopressor use	42 (44.2)	27 (22.9)	38 (37.7)	33 (32.2)

RR ≥ 25/min or mechanically ventilated	63 (66.3)	60 (50.8)	59 (58.5)	55 (53.8)

AMS	31 (32.6)	41 (34.7)	33 (33)	33 (33)

Other anti-MRSA agent^c^	19 (20)	21 (17.8)	22 (21.8)	21 (20.6)

Source of infection	43 (45.3)	47 (39.8)	46 (46.1)	38 (37.1)
Primary	39 (41.1)	55 (46.6)	40 (40.2)	52 (51.6)
Secondary	13 (13.7)	16 (13.6)	14 (13.7)	11 (11.3)
Catheter				

Foci of infection	20 (21.1)	29 (24.6)	21 (21.3)	21 (21)
Catheter	17 (17.9)	13 (11)	14 (14.2)	11 (10.8)
Vertebral bone/joint	23 (24.2)	28 (23.7)	24 (24.3)	24 (24)
Nonvertebral bone/joint	39 (41.1)	45 (38.1)	41 (40.8)	41 (40.1)
SSTI/Surgical	27 (28.4)	44 (37.3)	34 (33.5)	35 (35)
Endovascular	31 (32.6)	15 (12.7)	22 (22.2)	19 (18.8)
Respiratory	16 (16.8)	24 (20.3)	17 (16.9)	19 (18.9)
Hardware				

Source control^d^	86 (90.5)	103 (87.3)	92 (91.2)	91 (89.8)

Time to initial anti-MRSA agent from index blood culture collection, hours	4.75 + 2.27	10.44 + 1.45	7.46 + 2.19	9.03 + 1.25

Anti-MRSA agent started before index blood culture collection	13 (13.7)	10 (8.5)	13 (12.6)	12 (12.3)

Time to MRSA result from initial anti-MRSA agent administration, hours	16.31 + 2.43	15.35 + 1.61	14.83 + 2.08	16.74 + 1.71

Abbreviations: AMS, altered mental status; CKD/ESRD, chronic kidney disease/end stage renal disease; ICU, intensive care unit; qPitt, Pitt bacteremia score; RR, respiratory rate; SBP, systolic blood pressure; SSTI: skin and soft tissue infection.

a Data are presented as No. (%) for dichotomous variables and mean + standard deviation for continuous variables.

b Quan variant(28), non-age adjusted.

c Other anti-MRSA agent included rifampin, clindamycin, linezolid, and sulfamethoxazole/trimethoprim.

d Includes partial source control.

**Table 2:** Unweighted and propensity-score weighted analysis of the primary outcome and its individual components

Outcome	Unadjusted Analysis^a^		Unweighted regression	Propensity score-weighted regression
	Combination	Monotherapy	OR (95% CI)	OR (95% CI)
Primary Composite	41/95 (43)	29/118 (25)	2.23 (1.24, 4.01)	1.58 (0.60, 4.18)
Persistent bacteremia	18/95 (19)	8/118 (7)	--	4.28 (1.64, 11.19)
30-day mortality	25/95 (26)	21/118 (18)	--	1.00 (0.33, 3.08)
30-day recurrence	1/95 (1)	2/118 (2)	--	Not included^b^

a Data are presented as No. (%).

b Not included as too few patients had 30-day recurrence and therefore the model was unstable.

**Table 3:** Unweighted and propensity-score weighted analysis of secondary outcomes

Outcome	Unadjusted Analysis^a^		Propensity score-weighted regression
	Combination	Monotherapy	OR (95% CI)
Time to microbiological cure, days	4.4 + 3	2.8 + 2.4	Mean difference (95% CI) 1.50 (0.60, 2.41)
Hospital length of stay, days	28.8 + 37.9	17.4 + 17.9	Mean difference (95% CI) 13.38 (2.43, 24.34)
30-day readmission	33/99 (33)	20/69 (29)	0.74 (0.35, 1.54)
90-day recurrence	4/70 (5.7)	9/97 (9.3)	0.59 (0.51, 2.40)

a Data are presented as No. (%) for dichotomous variables and mean + standard deviation for continuous variables.

**Table 4:** Unweighted and propensity-score weighted analysis of safety Outcomes

Adverse drug event	Combination^a^ (n = 95)	Monotherapy^a^ (n = 118)	Propensity score-weighted regression OR (95% CI)

Acute kidney injury	28/71 (39)	38/104 (37)	1.49 (0.60, 3.34)
KDIGO Stage 1	15/71 (21)	25/104 (24)	--
KDIGO Stage 2	7/71 (10)	10/104 (10)	--
KDIGO Stage 3	6/71 (8)	4/104 (4)	2.69 (0.60, 12.13)

Neutropenia	6/78 (8)	9/89 (9)	1.21 (0.37, 3.98)

Eosinophilia	20/78 (26)	20/90 (22)	1.45 (0.66, 3.19)

Elevated CPK	3/53 (6)	3/31 (10)	--

Rash	1/90 (1)	2/105 (2)	--

ADE leading to antibiotic change or discontinuation	9/95 (9)	9/118 (8)	1.10 (0.36, 3.32)

Abbreviations: ADE, adverse drug event; CPK, creatine phosphokinase

a Data are presented as No. (%).

## Data Availability

The data that support the findings of this study are not openly available as they constitute protected health information (PHI) protected under HIPAA. Data are located in IRB-approved controlled access data storage at the University of Virginia Health System.
